# The continuing transformation of *Acta Crystallographica Section E* and the launch of *IUCrData*

**DOI:** 10.1107/S2056989015021040

**Published:** 2016-01-01

**Authors:** William T. A. Harrison, Helen Stoeckli-Evans, Edward R. T. Tiekink, Luc Van Meervelt, Matthias Weil

**Affiliations:** a Department of Chemistry, University of Aberdeen, Meston Walk, Aberdeen AB24 3UE, Scotland; b Institute of Physics, University of Neuchâtel, Rue Emile-Argand 11, CH-2000 Neuchâtel, Switzerland; c Centre for Chemical Crystallography, Faculty of Science and Technology, Sunway University, 47500 Bandar Sunway, Selangor Darul Ehsan, Malaysia; d Chemistry Department, KU Leuven, Celestijnenlaan 200F, B-3001, Leuven (Heverlee), Belgium; e Institute for Chemical Technologies and Analytics, Division of Structural Chemistry, Vienna University of Technology, Getreidemarkt 9/164-SC, A-1060 Vienna, Austria

**Keywords:** crystallography, editorial, *Acta E*

The first step of the relaunch of *Acta Crystallographica Section E* in June 2014 heralded its transformation from *Structure Reports Online* to *Crystallographic Communications*. It introduced papers in the new *Research Communications* format: these are full reports designed to bring out the science behind a structure determination. We are pleased to say that an ever increasing number of authors are choosing to publish their work as *Research Communications*, many discussing related structures in a single publication, making the most of the opportunity to include extra tables and figures in the published papers to illustrate their results and enhance the discussion of the underlying science. Those authors whose papers have typically included a detailed discussion accompanied by insightful figures have naturally been quick to embrace the *Research Communications* format. The short *Data Reports* are, however, still popular with many authors. With a little extra effort, a good proportion of these papers can be developed for publication as *Research Communications*. Such submissions are identified at the prescreening stage and the Section Editors and Co-editors then work closely with authors to help them to convert their article to the longer format by expanding the text and sometimes by combining a series of single-structure reports.

The variety of compounds covered so far in *Research Communications* is vast, from peptides to POMs (polyoxidometalates) and includes MOFs, coordination polymers, cage compounds, calixarenes, grid-type structures, isotypic structures, minerals, a fullerene, a potential secondary explosive, herbicides, a helicene, a noble gas compound, intermetallic compounds, a variety of drugs and even a compound that may form on the surface of Mars. *Research Communications* have also given insights into a wide range of topics such as atomic thermal vibration behaviour, hydrogen and halogen bonding, polymorphism, the influence of co-ligands, counter-anions and conformation on structure, luminescent properties and the relationship between structural features and bioactivity. While the majority of crystals have been measured with Mo Kα or Cu Kα radiation, some studies have benefited from synchrotron and even neutron time-of-flight measurements and we have seen reports of structure analyses from X-ray powder diffraction data. Many authors also report complementary techniques. 

One of the stated aims of the *Acta Crystallographica Section E* relaunch is to regain indexing in the Science Citation Index. We are therefore delighted to announce that the journal is now included in Thomson Reuters’ new Emerging Sources Citation Index (ESCI) which was launched in November 2015. The benefit for authors and researchers is that a journal in ESCI is searchable, discoverable, and citable in the main Science Citation Index; authors and researchers will be able to get real-time insight into a journal’s citation performance while the content is considered for inclusion in other Web of Science collections. We hope that the final phase of the transformation of *Acta Crystallographica Section E* outlined below will herald early re-indexing of the journal in the full Science Citation Index.

The final phase in the relaunch is the separation of *Research Communications* and *Data Reports*. From 2016, only *Research Communications* will be published in *Section E*. This does not, however, spell the end for short structure reports. The IUCr is fully committed to the original concept of *Acta Crystallographica Section E*, *i.e.* a mechanism to ensure that solved and refined structures are not lost. There is still a real need for structures to get into the public domain after the scrutiny of peer review. From 2016, *Data Reports* will have a home in the IUCr’s new open-access data publication, *IUCrData*. *Data Reports* will be open access and peer reviewed as now, and they will each have an ID and DOI.

As part of the changes, the IUCr’s *publCIF* software has been upgraded. *publCIF* allows authors to write and edit papers using a word-processing environment, and is the preferred way to prepare submissions for *Acta E*. *publCIF* takes a crystallographic information file (CIF) and prepares a formatted paper (preprint) in the *Research Communication* style. The CIF and the preprint are presented side-by-side and can both be edited – changes made to one are applied to the other as you type. *publCIF* also includes many useful editorial tools and can be used to add data items required for publication, prepare standard and customized geometry tables, check the CIF for both syntax and completeness, print or export a preprint, check the references and more. Using *publCIF*, a *Research Communication* can be produced with little extra effort. Be sure to use the latest version of *publCIF*, which is available to download free of charge from http://publcif.iucr.org.

As always, the transformation of the journal and the success of the *Research Communication* format are down to our dedicated team of Co-editors who are doing an excellent job in advising authors how to promote their science. On behalf of the Section Editors of *Acta Crystallographica Section E*, we would like to take this opportunity to thank them, as well as our Editorial Advisory Board members and the staff in Chester for their continued help in making *Acta Crystallographica Section E* the obvious choice for disseminating the results of the excellent crystallographic studies that are being carried out by our authors worldwide.

We wish *IUCrData* a successful take-off. For more information, please visit its website at http://iucrdata.iucr.org.

